# Interaction of agonists of a different subtype of the nAChR and carvacrol with GABA in *Ascaris suum* somatic muscle contractions

**DOI:** 10.21307/jofnem-2021-022

**Published:** 2021-03-26

**Authors:** Djordje S. Marjanović, Saša M. Trailović, Mirjana Milovanović

**Affiliations:** Faculty of Veterinary Medicine, University of Belgrade, Bul. oslobodjenja 18, 11000, Belgrade, Serbia

**Keywords:** nAChR, GABA, Carvacrol, Interaction, Resistance

## Abstract

Resistance of parasitic nematodes to anthelmintic drugs is a growing problem in human and veterinary medicine. The molecular mechanisms by which nematodes become resistant are different, but certainly one of the possible processes involves changing the drug binding site on the specific receptor. The significance of changes in individual subtypes of nicotinic acetylcholine receptors (nAChRs) for the development of resistance has not been clarified in detail. This study investigates the interaction of antinematodal drugs, agonist of different types of nAChRs and carvacrol with gamma aminobutyric acid (GABA) on the contractions of parasitic nematode *A. suum*. In our study, GABA (3 μM) produced significant increase of contractile EC_50_ value for pyrantel, and nonsignificant for bephenium and morantel, from 8.44 to 28.11 nM, 0.62 to 0.96 µM, and 3.72 to 5.69 nM, respectively. On the other hand, the maximal contractile effect (*R*_max_) did not change in the presence of GABA. However, when *A. summ* muscle flaps were incubated with GABA 3 μM and carvacrol 100 μM, the EC_50_ value of pyrantel, bephenium, and morantel was increased significantly to 44.62 nM, 1.40 μM, and nonsignificantly to 7.94 nM, respectively. Furthermore, *R*_max_ decreased by 70, 60, and 65%. Presented results indicate that the combined use of GABA receptor agonists and nicotinic receptor antagonists can effectively inhibit the neuromuscular system of nematodes, even when one of the nicotinic receptor subtypes is dysfunctional, due to the potential development of resistance.

A number of excitatory and inhibitory neurotransmitters are involved in the regulation of somatic muscle contractions in nematodes. It is well known that excitatory and inhibitory neurons, whose coordinated activity ensures sinusoidal movement have an equivalent role in nematode locomotion. The excitation of somatic muscles is provided by acetylcholine (ACh), which leads to contraction. Cholinergic transmission occurs both between neurons and at the neuromuscular junction. Furthermore, the contraction of the ventral wall causes the dorsal wall to relax on the other side of the body. The relaxation of the somatic muscles takes place through GABA, and in this way the two neurotransmitters coordinate the movement of the nematode. Acetylcholine (ACh) is a neurotransmitter in the excitatory synapses, while GABA is the neurotransmitter in the inhibitory synapses between interneurons and motor neurons. These interneurons could be related evolutionarily to mammalian Renshaw cells, and are potentially a very attractive drug target (Trailovic and Varagic, 2007).

The most commonly used antinematodal drugs in human and veterinary medicine are cholinergic agonists: imidazothiazole (levamisole), tetrahydropyrimidines (pyrantel, morantel and oxantel), quaternary/tertiary amines (bephenium and tribendimidine), pyridines (methyridine), and amino-acetonitrile derivatives (AADs; monepantel). This group of drugs produces spastic paralysis of the nematode by selectively gating acetylcholine receptor ion-channels at the synapses of motor neurons and interneurons, or at somatic muscle cells. The result of opening of the sodium ion channel is depolarization, followed by a contraction (Holden-Dye et al., 1989). Pharmacologically, the nematode muscle nicotinic acetylcholine receptors (nAChRs) have similarities to the vertebrate neuronal receptor, because it is relatively insensitive to block by bungarotoxin (Tornøe et al., 1995). Unlike the vertebrates, there is more than one type of nAChR found in the musculature of adult nematodes. In *Ascaris suum*, there are at least three types of nAChR located on the muscle, with distinguishable pharmacological and single-channel properties. Each of the three subtypes was named based on its preferred agonist, N (prefers nicotine and oxantel), L (prefers levamisole and pyrantel), and B (prefers bephenium) (Martin et al., 2004; Robertson et al., 2002).

Another large group of antinematodal drugs are avermectins. Avermectins interact with the gamma aminobutyric acid (GABA) receptor in somatic muscle, and the glutamate-gated chloride channel, which exists only in the pharynx of nematodes. This interaction leads to increased permeability of the postsynaptic membrane to chloride ions, causing hyperpolarization of the membrane and muscle relaxation, or atonic paralysis and death of parasites (Holden-Dye and Walker, 2005-2018; Puttachary et al., 2013; Wolstenholme, 2011).

In addition to receptors on nerve-muscle synapses, it was proved that functional GABA and nicotinic receptors exist extrasynaptically in the bag region of *Ascaris* muscle cells (Martin, 1982). These are the reasons why a large number of antinematodal drugs act on the nicotinic or GABA receptor and thus disrupt the locomotion of the parasite, which ultimately leads to death.

Carvacrol is a phenolic monoterpenoid, present as a secondary metabolite in many plant essential oils, such as thyme oil and oregano oil. Evidence of antinematodal effects of carvacrol has been published both in vivo and in vitro experiments. Our previous studies have shown that carvacrol is most likely a non-competitive antagonist of nAChRs in parasitic nematodes (Marjanović et al., 2020; Trailović et al., 2015). Furthermore, Lei et al. (2010) showed that carvacrol and thymol at concentrations of 330 μM caused an *A. suum* mortality rate of 80% (in vitro test during 24 hr).

We considered it important to examine the effect of antinematodal drugs, agonists of different types of nAChRs and carvacrol with GABA on the contractions of the parasitic nematode *A. suum*. This study could indicate the possibility of using two or more antinematodal drugs with different mechanisms of action in order to neutralize the resistance of the parasite.

## Materials and methods

### A. suum

Adult females of *Ascaris suum* were collected at the Ambar slaughterhouse in Surčin, Serbia and maintained in Locker solution (mM): NaCl 155, KCl 5, CaCl_2_ 2, NaHCO_3_ 1.5, and glucose 5, at a temperature of 32°C. The locker solution was changed twice a day. After collection, the worms were used for contractions assay for the next four days. *Ascaris* muscle flaps for contractions were prepared as previously described (Trailović et al., 2016). Each flap (always the same length of 1 cm) was monitored isometrically by attaching a force transducer in an experimental bath maintained at 37°C, containing 20 ml *Ascaris* Perienteric Fluid Ringer/APF-Ringer (mM): NaCl, 23; Na-acetate, 110; KCl, 24; CaCl_2_, 6; MgCl, 5; glucose, 11; HEPES, 5; pH 7.6, and bubbled with room air. After dissection, the preparations were allowed to equilibrate for 15 min under the initial tension of 0.5 g. Different concentrations of pyrantel, morantel, and bephenium were then added to the preparation, and the maximum contraction was observed before the washing and subsequent application of the next concentration of the drug. The responses for each concentration were expressed in grams (g), produced by each individual flap preparation. The effects of GABA (3 μM), and GABA + carvacrol 100 μM on control dose-response plots were determined. Contractions were monitored on a PC computer, using a BioSmart interface, and eLAB 44 software (ElUnit, Belgrade). Sigmoidal concentration-response curves for each agonist in the absence or presence of antagonist were described by the Hill equation.

### Drugs

Carvacrol, morantel, pyrantel, bephenium, and GABA were obtained from Sigma-Aldrich Co (St Louis, MO, USA). Morantel, pyrantel, bephenium, and GABA were dissolved in the APF-Ringer, while carvacrol was dissolved in ethanol, with the final concentration of ethanol in the APF-Ringer of 0.1%v/v. When tested, 0.1% of ethanol did not alter the resting activity of preparations and did not alter the drug responses.

### Data analysis

In the studies presented here, sigmoid concentration dose–responses were described by the equation: % response = 1/1 + [EC50/Xa]nH, where the median effective concentration (EC_50_) is the concentration of the agonist (Xa) producing 50% of the maximum response and nH is the Hill coefficient (slope). Prism 6.0 (GraphPad Software, San Diego, California, USA) was used to estimate the constants EC_50_ and nH, by non-linear regression for each preparation. We determined mean contraction responses to each concentration of ACh (control dose-response: CR[Ach]) and mean responses to each of the concentrations of ACh in the presence of GABA. One-way analysis of variance (ANOVA) was applied for the analysis of the differences between the EC_50_ value and the *R*_max_ (maximal effect). Differences were considered significant when the *p* value was < 0.05. The statistical analysis was done using Graphpad Prism software, while all values are expressed as mean ± standard error (S.E.).

## Results

**Figure 1:**
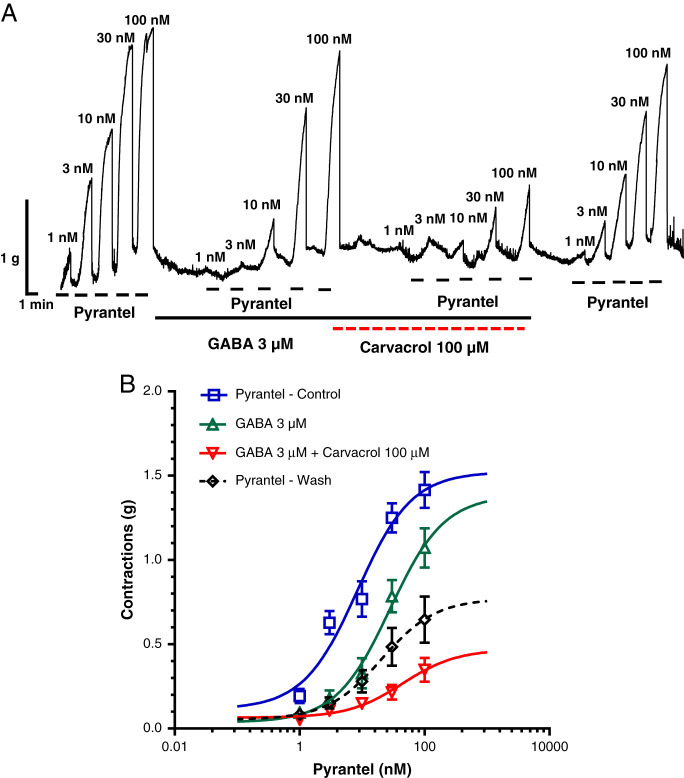
A Original recording of isometric contractions of *A. suum* muscle flap induced by increasing concentrations of pyrantel and the effect of GABA (3 μM) and Carvacrol (100 μM) on those contractions; B the concentration-response plot for pyrantel control (*n* = 6, blue), in the presence of GABA 3 μM (*n* = 6, green), in the presence of GABA and Carvacrol 100 μM (*n* = 6, red) and after washing (*n* = 6, dashed black) (mean ± S.E.).

In our study, pyrantel caused concentration-dependent contractions of neuromuscular preparation of *A. suum* (Fig. 1A). After incubation with 3 μM of GABA, the EC_50_ of pyrantel increased significantly from 8.44 to 28.22 nM (Table 1). However, when the muscle flaps were incubated with GABA 3 μM and carvacrol 100 μM at the same time, the EC_50_ of pyrantel reached as much as 44 nM, which is significantly higher than the control value and the EC_50_ of pyrantel after incubation with GABA 3 μM (Table 1). Part of this inhibitory effect is reversible, as after washing, the EC_50_ value of pyrantel returned to the level observed in the presence of GABA 3 μM, but did not reach the initial control level. On the other hand, the value of the maximum contractile effect (*R*_max_) of pyrantel has not changed in the presence of GABA 3 μM, but decreased significantly after incubation with combination of GABA and carvacrol (Table 2 and Fig. 1B).

**Table 1.** EC_50_ (±S.E.) values of pyrantel, bephenium, and morantel in the presence of GABA and carvacrol.

	Control	GABA 3 μM	GABA 3 μM + Carvacrol 100 μM	Wash
Pyrantel (nM)	8.44 ± 1.56	28.11 ± 1.61****	44.62 ± 2.70****^++++^	20.51 ± 2.34***^oooo^
Bephenium (μM)	0.62 ± 0.11	0.96 ± 0.21	1.40 ± 0.12***	1.32 ± 0.09**
Morantel (nM)	3.72 ± 1.73	5.69 ± 2.12	7.94 ± 2.35	15.81 ± 4.03*^+^

**Notes:** *Statistically significant difference compared to control (***p < 0.0001; ***p = 0.0007; ***p = 0.0008; **p = 0.0032; *p = 0.0108); ^+^statistically significant difference compared to GABA 3 μM (^++++^ *p* < 0.0001; ^+^ *p* = 0.0429); ostatistically significant difference compared to GABA 3 μM + Carvacrol 100 μM (^oooo^*p* < 0.0001).

**Table 2.** *R*_max_ (g ± S.E.) values of pyrantel, bephenium, and morantel in the presence of GABA and carvacrol.

	Control	GABA 3 μM	GABA 3 μM + Carvacrol 100 μM	Wash
Pyrantel	1.52 ± 0.12	1.38 ± 0.20	0.47 ± 0.15****^+++^	0.77 ± 0.17**^+^
Bephenium	2.20 ± 0.30	2.18 ± 0.34	0.89 ± 0.22**^++^	1.15 ± 0.23*^+^
Morantel	1.16 ± 0.07	0.99 ± 0.11	0.39 ± 0.11****^+++^	0.59 ± 0.15***^o^

**Notes:** *Statistically significant difference compared to control (****p < 0.0001; ***p = 0.0007; **p = 0.0448; **p = 0.0062; *p = 0.0475); ^+^**s**tatistically significant difference compared to GABA 3 μM (^+++^*p* = 0.0008; ^+^*p* = 0.0429; ^++^*p* = 0.0072; ^+^*p* = 0.0475; ^+++^*p* = 0.0003); ^o^statistically significant difference compared to GABA 3 μM + Carvacrol 100 μM (^o^*p* = 0.0298).

**Figure 2:**
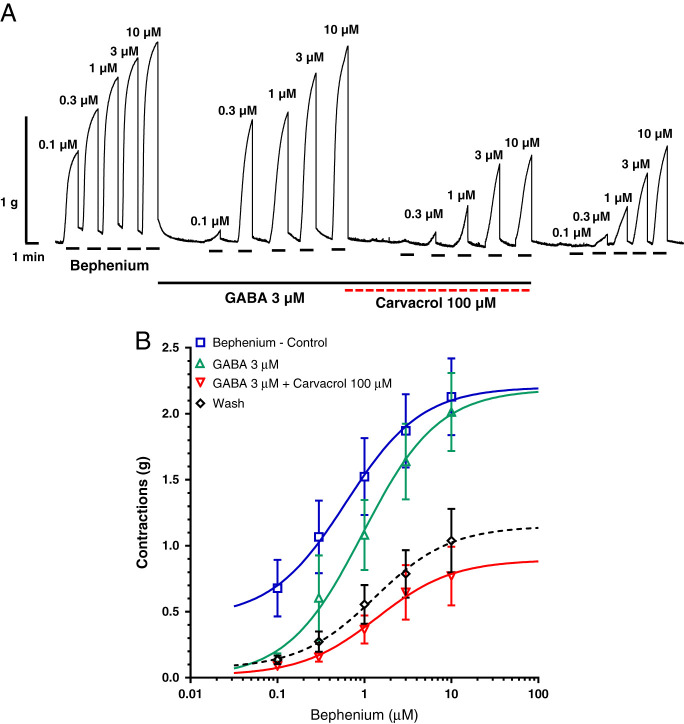
A Original recording of isometric contractions of *A. suum* muscle flap induced by increasing concentrations of bephenium and the effect of GABA (3 μM) and Carvacrol (100 μM) on those contractions; B the concentration-response plot for bephenium control (*n* = 6, blue), in the presence of GABA 3 μM (*n* = 6, green), in the presence of GABA and Carvacrol 100 μM (*n* = 6, red) and after washing (*n* = 6, dashed black) (mean ± S.E.).

The control value of the bephenium EC_50_ was 0.62 μM and it increased nonsignificantly to 0.96 μM in the presence of GABA 3 μM (Table 1). A representative recording of contractions caused by bephenium and the sigmoid concentration dose–responses plot are shown in Figure 2A, B. Furthermore, the EC_50_ value of bephenium reached a level of 1.44 μM in the presence of GABA and carvacrol, which is highly significant compared both to the control and the EC_50_ of bephenium obtained in the presence of GABA (Table 1). Something different was obtained with the maximum contractile effect of bephenium (*R*_max_), because it did not change in the presence of GABA. However, the combination of GABA and carvacrol significantly reduced the *R*_max_ value from 2.2 to 0.89 g (Table 2).

**Figure 3:**
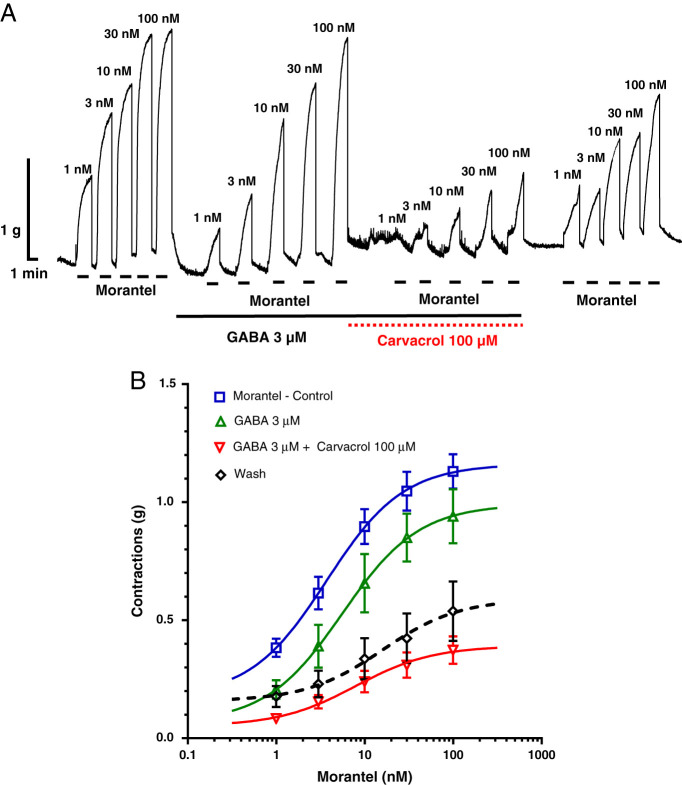
A Original recording of isometric contractions of A. suum muscle flap induced by increasing concentrations of morantel and the effect of GABA (3 μM) and carvacrol (100 μM) on those contractions; B the concentration-response plot for morantel control (*n* = 6, blue), in the presence of GABA 3 μM (*n* = 6, green), in the presence of GABA and carvacrol 100 μM (*n* = 6, red) and after washing (*n* = 6, dashed black) (mean ± S.E.).

The EC_50_ of morantel did not change significantly in the presence of GABA and in the presence of a combination of GABA and carvacrol (morantel EC_50_ = 3.72, 5.69, and 7.94 nM) (Table 1). Moreover GABA 3 μM did not change the *R*_max_ of morantel either, but the combination of GABA and carvacrol significantly reduced the value of the maximal contractile effect (Table 2). Figure 3 shows a representative recording of *A. suum* muscle strip contractions caused by morantel (Fig. 3A) and sigmoid concentration dose–responses plot (Fig. 3B).

The applied concentration of GABA (3 μM) did not cause relaxation of the tested muscle strips.

## Discussion

We believe that the concept of simultaneous use of compounds with multiple sites of antiparasitic action is able to prevent the development of resistance in parasites. We have shown in previous studies that carvacrol is most likely to act as a non-competitive inhibitor of nAChRs of nematodes (Marjanović et al., 2020; Trailović et al., 2015). It is known that one of the mechanisms for the development of resistance involves a change in the receptor to which the drug binds (Abongwa et al., 2017). For example, it is known that pyrantel resistance is associated with a modification of the target nicotinic receptor properties (Robertson et al., 2000). Whether this can also refer to the change of only certain subtypes of the nicotinic receptor has not been clarified.

Pyrantel is an anthelmintic from the tetrahydropyrimidine group. It is used in veterinary and human medicine against gastrointestinal nematodes. Pyrantel is a fairly selective agonist of nAChRs. It produces depolarization and contraction of the nematode body muscle which causes spastic paralysis. Pyrantel is a relatively potent agonist (EC_50_ ~ 10 µM) on levamisole receptor subtypes (L-subtype) of nematode nAChRs. Our results are in accordance with the presented data. In our study, the contractile EC_50_ of pyrantel for *A. suum* muscle flaps was 8.44 μM, and it increased more than threefold in the presence of GABA, without altering the *R*_max_. Such an inhibition is manifested by competitive antagonists, but GABA certainly is not a competitive antagonist of nAChRs. We previously obtained a similar result with ACh and hypothesized that this phenomenon occurs as a consequence of the binding of GABA, in low concentrations, to the GABA receptor in the synapses between inhibitory interneurons and motoneurons (synaptic effect). This process immediately activates the competitive antagonistic excitatory mechanism through excitatory interneurons (Trailović et al., 2015). On the other hand, this study shows that the addition of carvacrol had a non-competitive antagonistic effect because the *R*_max_ was reduced by more than three times. We hypothesize that carvacrol cannot bind presynaptically but rather postsynaptically to a different site on nAChR than pyrantel, resulting in non-competitive antagonism.

Bephenium is an anthelmintic compound used against human and dog hookworms and gastrointestinal parasitic nematodes in sheep (Burrows, 1958). Bephenium activates the B-subtype of the nAChRs of nematodes (Martin and Robertson, 2007), but there is no more detailed information on the mechanism of action. In our study, GABA did not alter the EC_50_ of bephenium, nor did it affect the maximal contractile effect (*R*_max_). Furthermore, the addition of carvacrol led to a significant increase in bephenium EC_50_ but also a decreased the *R*_max_. This is obviously mostly non-competitive antagonism and can be explained by the fact that carvacrol binds to a different site on the receptor relative to the binding site of bephenium, and that the B-subtype of nAChRs are predominantly located postsynaptically.

The results obtained with morantel are especially interesting. Morantel also belongs to tetrahydropyrimidines, but it is still not clear which subtype of nAChRs it predominantly prefers. The incubation of the muscle strips with GABA did not significantly change the EC_50_ of morantel, nor the *R*_max_ value. There was no significant change in EC_50_ of morantel even after the incubation with the combination of GABA + carvacrol, but the *R*_max_ was significantly reduced. Similar to bephenium, we can state that the inhibitory effect of GABA is almost absent, while the combination of GABA and carvacrol exhibits mostly non-competitive antagonism. As before, we could assume that the receptors to which morantel binds are predominantly localized postsynaptically, which causes the absence of activation of inhibitory interneurons, and consequent competitive response. Courtot et al. (2015) reported that ACR-26 and ACR-27, the nAChRs subunits from *Parascaris equorum* expressed in *Xenopus* oocyte, are able to form a functional nAChR highly sensitive to pyrantel and morantel.

In our previous study (Trailović et al., 2015), GABA 3 μM shifted the acetylcholine EC_50_ to the right, producing a dose-ratio (EC_50control_/EC_50GABA_) of 2.39, while the *R*_max_ was reduced by 30%. Now, after the incubation with 3 μM of GABA the dose ratios were 3.33 for pyrantel, 1.54 for bephenium, and 1.60 for morantel. On the other hand, *R*_max_ for pyrantel, bephenium, and morantel was reduced by 9, 0.1, and 14.6%, respectively. It is obvious that GABA most effectively inhibits the effect of pyrantel and that the majority of this effect is competitive. We assume that this is the result of the synaptic effect in the synapses between inhibitory interneurons and motoneurons. However, when GABA was combined with carvacrol the dose-ratio (EC_50control_/EC_50GABA + carvacrol_) was 6.93, 2.25, and 2.13 for pyrantel, bephenium, and morantel, respectively, while *R*_max_ decreased by 70, 60, and 65%, respectively. Their effect is more dominantly non-competitive, but also indicates that the combined use of GABA receptor agonists and nicotinic receptor antagonists can effectively inhibit the neuromuscular system of nematodes, even when one of the nicotinic receptor subtypes is dysfunctional due to the development of resistance. This is useful information for the formulation of commercial drugs that could prevent and overcome resistance.
